# The RAVI registry: prospective, multicenter study of radial access in embolization procedures – 30 days follow up

**DOI:** 10.1186/s42155-023-00415-5

**Published:** 2024-01-30

**Authors:** Marcelo Guimaraes, Aaron Fischman, Hyeon Yu, Jordan Tasse, Jessica Stewart, Keith Pereira, for the RAVI Registry Investigators, Andre Uflacker, Ricardo Yamada, Kirubahara Vaheesan, Ali Abdullah Malik, Afsheen Sherwani, Clayton Commander, Maureen Kohi, Robert Lookstein, Francis Nowakowski, Rahul Patel, Rajesh Patel, Joseph Titano, Mona Ranade

**Affiliations:** 1 Department of Radiology and Radiological Science, Medical University of South Carolina, 25 Courtenay Drive MSC 226, Room 3304, Charleston, SC 29425 USA; 2 Department of Diagnostic, Molecular and Interventional Radiology, Urology and Surgery, Icahn School of Medicine, Mt. Sinai Hospital, New York City, NY USA; 3 Department of Radiology, Division of Interventional Radiology, University of North Carolina, Chapel Hill, NC USA; 4 Department of Diagnostic Radiology and Nuclear Medicine, Division of Interventional Radiology, Rush University Medical College, Chicago, IL USA; 5 Department of Radiology, Division of Interventional Radiology, David Geffen School of Medicine, University of California Los Angeles, Los Angeles, CA USA; 6 Division of Interventional Radiology, St. Louis University School of Medicine, St. Louis, MO USA

## Abstract

**Background:**

There is a lack of registry studies about transradial access (TRA) outcomes. This prospective registry evaluated the TRA and procedure outcomes of visceral embolizations performed via TRA with 30-day follow-up.

**Material & methods:**

Prospective, multicenter registry included uterine fibroids (UFE), prostate artery (PAE), liver tumors (LT), and other hypervascular tumors (OHT) embolization performed in six US hospitals. Between February 2020 and January 2022, 99 patients underwent one radial artery visceral intervention (RAVI); 70 had UFE (70.7%), 16 PAE (16.2%), 7 LT (7.1%), and 6 OHT (6.1%). The mean age was 50.1 (±11.1) years, and 74/99 (74.7%) were females. The primary safety endpoints included hand ischemia, stroke, and death. Procedural success was defined as completing the intended procedure via radial artery (RA) access. Technical success was defined as the successful delivery of HydroPearl™ microspheres and complete embolization of the target vessel.

**Results:**

Procedural and technical successes were 100% and 97%, respectively. There was no stroke, hand ischemia, radial-to-femoral conversion, access-related serious adverse events, or clinically evident radial artery occlusion at 30 days. There were two deaths: one respiratory failure and one progression of liver disease. Minor RA-related adverse event included arterial spasm, hematoma, and post-procedure discomfort.

**Conclusion:**

This prospective, multicenter, open-label registry confirmed the high safety profile and effectiveness of radial access in UFE, PAE, LT, and OHT embolization procedures without stroke, hand ischemia, or access-related serious adverse events at 30-day follow-up.

## Background

The scientific evidence to support the use of transradial access (TRA) in interventional cardiology has progressively increased in the last 30 years and is now widely accepted worldwide. In 2015, the European Society of Cardiology Guidelines and then in 2021, the Report of the American College and American Heart Association Joint Committee on Clinical Practice Guidelines recommended radial access over transfemoral access (TFA) (IA recommendation) for coronary angiography and interventions [1, 2]. In the last decade, interventional radiologists have slowly adopted TRA, especially for embolization procedures. Several case series showed the efficacy and safety of radial access in visceral interventions (RAVI) [3–6]. Other prospective, randomized studies comparing TFA versus TRA in uterine fibroid embolization (UFE), prostate artery embolization (PAE), embolization of liver cancer, and splenic trauma have demonstrated that TRA can have equivalent outcomes or be superior to TFA [7–12]. Iezzi et al. described the perception of the IR community in Europe and in the USA about TRA and the potential limiting factors for the adoption of this technique [13]. With the aim to study radial access outcomes prospectively and without a controlled methodology, to address some skepticism about potential TRA-related adverse events and the lack of “real-world” data on RAVI, we conducted a registry study to evaluate the outcomes of embolization procedures performed via TRA with 30 days follow-up.

## Material and methods

### Study design

The RAVI registry (ClinicalTrials.gov Identifier: NCT04272216) was a prospective, multicenter, open-label study with patients who were sequentially enrolled without randomization. The goal was to enroll up to 100 subjects in total, with no more than 30% of the population from any given site. It was a sponsored study performed by interventional radiologists with experience in RAVI and included a total of six different medical centers through a research grant provided to each site (Terumo Medical Corporation, Somerset, New Jersey/USA). All patients provided written informed consent before inclusion. Data was collected prospectively from electronic medical records and monitored by local investigators and research support members. Institutional Review Board approval was independently obtained by every site. For this analysis, we included data from the procedure day, from the period of hospital admission (for the patients who required hospitalization post-procedure), and from the 30-day follow-up clinic visit.

### Study population

Between February 2020 and January 2022, 105 patients were screened, and 99 enrolled patients had elective embolization procedures in six different academic medical centers: 70 UFE (70.7%), 16 PAE (16.2%), 7 LT (7.1%) and 6 OHT (6.1%). The cohort mean age was 50.1 (±11.1) years old, and 74/99 (74.7%) were females (Table 1). Most patients were African American (49.5%) or Caucasian. (42.4%) (Table 1). The most common comorbidities were hypertension (43.4%) and drug allergies (37.4%) (Table 2). The inclusion criteria included any patient 18 years or older, of any gender, who had hypervascular benign or malignant tumors, who met the criteria for TRA, and was considered eligible for the study. TRA eligibility criteria included a positive Barbeau test [14] and pre-procedure ultrasound (US) to check patency and whether the RA had > 1.6 mm in the antero-posterior diameter. The patient needed to be able to provide informed consent and be willing to participate in the 30-day clinical follow-up for the primary endpoint. The exclusion criteria included typical exclusions for angiographic procedures, such as female patients of childbearing potential who were pregnant, not taking adequate contraceptive measures, or breastfeeding. Conditions precluding TRA included extensive arterial calcifications, Barbeau D or radial artery too small to safely accommodate an introducer sheath. Other exclusion criteria included current participation in another clinical trial that would interfere with the study endpoints, patients with a history of hemorrhagic and/or ischemic stroke, and patients with planned surgical intervention to the wrist/forearm within 30 days after the study procedure. Distal radial, ulnar, or femoral artery accesses were excluded from the study.

**Table 1:** Patient demographics

Demographics	UFE (***n*** = 70)	PAE (***n*** = 16)	LT (n = 7)	OHT (***n*** = 6)	All Subjects (***n*** = 99)
Mean Age (year) ± SD	44.3 ± 5.3	67.6 ± 7.8	59.6 ± 9.2	60.3 ± 6.8	50.1 ± 11.1
Female	100%	0.0%	14.3%	50.0%	74.7%
**Race**					
Native American or Alaskan	1.4%	0.0%	0.0%	0.0%	1.0%
Asian	1.4%	0.0%	0.0%	0.0%	1.0%
Black or African American	62.9%	6.3%	42.9%	16.7%	49.5%
Pacific Islander	0.0%	0.0%	0.0%	0.0%	0.0%
Caucasian	27.1%	87.5%	57.1%	83.3%	42.4%
Other	7.1%	6.3%	0.0%	0.0%	6.1%

**Table 2:** Medical comorbidities

Medical History	UFE (n = 70)	PAE (n = 16)	LT (n = 7)	OHT (n = 6)	All Subjects (n = 99)
Myocardial Infarction	1.4%	6.3%	0.0%	0.0%	2.0%
Atrial Fibrillation	0.0%	25.0%	0.0%	0.0%	4.0%
Drug Related Allergies	38.6%	37.5%	42.9%	33.3%	37.4%
Hypertension	37.1%	43.8%	57.1%	100%	43.4%
Hypercholesterolemia	4.3%	25.0%	14.3%	50.0%	11.1%
Liver Disease	1.4%	0.0%	100%	50.0%	11.1%
Extrahepatic Disorders	1.4%	0.0%	28.6%	33.3%	5.1%
Prostate Disease	0.0%	100%	0.0%	0.0%	16.2%

### Technical considerations

A pulse oximeter was placed on the left thumb or index finger and maintained throughout the procedure and recovery time to monitor the hand perfusion. Most procedures were conducted under intravenous moderate sedation.

Just prior to prepping the patient’s forearm for RA, the puncture target zone (1–2 cm proximal from the styloid process) was evaluated with US to check for patency and calcifications. If no calcifications were present at the puncture site, then TRA was obtained. No score system was used to judge RA calcifications. Local anesthesia was administered to the left wrist using a small amount (~ 1 mL) of 1% lidocaine. TRA was obtained under US guidance [15]. A TRA kit (Glidesheath Slender®, Terumo Medical Corporation, Somerset, New Jersey/USA), which includes a short 21-gauge needle, a 0.021″ nitinol wire, and a 4, 5 or 6 French 10-cm long hydrophilic coated introducer sheath, was used to obtain RA. The sheath diameter selection was at the operator’s discretion. Once TRA was established, medications to prevent vasospasm and clot formation were used at the operator’s discretion. At least 200 mcg of Nitroglycerine, with or without 2.5 mg of Verapamil was administered to prevent spasms, and at least 3000 IU of unfractionated Heparin was used for clot prevention [15].

To obtain access from the left wrist to the descending aorta, a 0.035″ 150 cm hydrophilic J Glidewire® was used in combination with a diagnostic catheter (5-Fr 110 cm long Optitorque Sarah, 5-Fr 110 cm long Optitorque Jacky, or 5-Fr 125 cm long Glidecath® hydrophilic R.A.V.I. MG1; Terumo Medical Corporation, Somerset, New Jersey/US). The selection of microcatheter and spherical embolic particle sizes for the selective embolization depended on the type of procedure and the operator’s preference. A 2.0, 2.4 or 2.8-Fr PROGREAT® microcatheter (Terumo Medical Corporation, Somerset, New Jersey/USA) was used for the infusion of microspheres with sizes varying between 200 to 800 μm (HydroPearl™, Terumo Medical Corporation, Somerset, New Jersey/US). At the end of the procedure, a TR Band™ radial compression device (Terumo Medical Corporation, Somerset, New Jersey/USA) was used to obtain RA hemostasis using patent hemostasis technique [16–18].

### Post-procedure care

In the recovery area, the patients were monitored with a continuous pulse oximeter kept on the same left index finger or thumb, and the left upper extremity was evaluated (for hand pain, tenderness, weakness, or sensory deficit, and skin color/temperature changes) before discharge [17]. The TR Band™ was removed according to its standard instructions for use. The patients were discharged after a brief period of observation of 15–20 minutes after the removal of the TR Band™. They were oriented to return to the emergency department or to contact the physician provider or the research team in case there was any post-embolization syndrome, abnormality in the left hand/wrist, or any neurological deficits. Some patients required overnight stay to control post-embolization syndrome (abdominal/pelvic pain, nausea, low-grade fever, vomiting). None of the patients had anticoagulation or antiaggregation medications started after the procedure day. All patients had follow-up evaluations in the clinic between 4- and 5-weeks post-intervention.

## Definitions

Procedure success was defined as completing the planned procedure via TRA without conversion to TFA. Technical success was defined as having effectiveness in the embolization of the target vessel until stasis (i.e., complete embolization). The “adverse events” (AE) were divided into serious and non-serious. Serious AE (SAE) was defined as an outcome of a procedure resulting in a life-threatening event, prolonged patient hospitalization or re-hospitalization after being discharged home, disability or permanent damage, intervention or treatment to prevent permanent impairment or damage, or death. Non-serious AE was defined as any AE that does not fulfill the definition of an SAE [19]. In this registry, examples of non-serious AEs were post-embolization syndrome (abdominal pain, nausea) controlled with oral medication and did not require hospital readmission, prolonged admission, or any further work-up. Hematoma is defined as the presence of visible self-limited blood extravasation, with variable extension along the forearm, not associated with any nerve deficit and that did not require surgical evacuation. Clinically relevant radial artery occlusion is defined as symptomatic total obstruction of the artery, typically by thrombus, usually at the site of access requiring anticoagulation or surgical/endovascular repair. Peripheral embolization is defined as a loss of distal pulse/perfusion, pain, and/or discoloration of the fingers. Hand ischemia is defined as having post procedure hand skin color changes and/or patient’s complaint about hand pain or motor/sensory deficit. Radial artery vasospasm is defined as the contraction of radial artery which was documented by the initial forearm angiogram finding of reduction in caliber of the radial artery that could be focal or segmental. Dissection is defined as a disruption of an arterial wall resulting in the splitting and separation of the intimal layers. Pseudoaneurysm is defined as disruption and dilation of the arterial wall without identification of the arterial wall layers at the site of the catheter entry demonstrated by arteriography or ultrasound. Arteriovenous fistula is defined as a connection between the access artery and the accompanying vein that is demonstrated by arteriography or ultrasound. Symptomatic stroke was defined as the presence of any new neurological deficit reported by the patient. Time to hemostasis was defined as the time between TR Band™ insufflation on the TRA until it was removed without any bleeding at the puncture site. Time to ambulation was defined by the length of time between the end of the procedure and when the patient was able to get out of bed post-procedure. Time to discharge was defined by the length of time between the end of the procedure and when the patient went home. Procedure length was defined by the length of time from administering a local anesthetic to the wrist until hemostasis obtained at the RA puncture site with the TR Band™. Patent hemostasis technique was defined as having enough balloon compression on the radial artery access to simultaneously prevent RA bleeding while keeping the radial artery patent. The introducer sheath was pulled out 4–5 cm and a plastic wrist band was placed around the forearm at the site of entry. A gauze composite was placed over the site of entry. A pulse oximeter sensor was placed over the index finger, the wrist band balloon was insuflated with 15 cc of air, and the sheath was removed. The balloon was slowly deflated until bleeding was seen through the arteriotomy, when 2 cc of air was reinflated. The ipsilateral ulnar artery was manually compressed. If the plethysmographic signal returned (confirming RA patency), then nothing else was done. In case there was signal, then 1 cc out of the 2 cc of reinflated air was removed and the ipsilateral ulnar artery was manually compressed again. If the plethysmographic signal returned (confirming RA patency), then patent hemostasis was obtained and nothing else was done. The wrist band was left in place for 2 hr.

### Endpoints

The registry endpoints focused primarily on procedure safety and effectiveness. The primary safety endpoints were death, myocardial infarct, symptomatic stroke, and hand ischemia. The secondary safety endpoints were divided in access-related immediate/trans-procedure (RA spasm or dissection, forearm artery perforation) and TRA-specific adverse events within 30 days (hematoma at the puncture site, symptomatic RA occlusion, arteriovenous fistula, pseudoaneurysm, distal embolism to the digits, finger amputation/loss of limb or any adverse events requiring surgical and/or endovascular intervention within 30 days of index procedure). Primary effectiveness endpoints included procedure success, conversion from TRA to TFA, and technical success. Secondary effectiveness endpoints included time to hemostasis, time to ambulation, time to discharge, procedure length, contrast volume, and blood loss > 5 mL.

### Statistical analysis

All statistical analyses were conducted using SAS version 9.4 (SAS Institute Inc., Cary, NC). To minimize data analysis bias, the data was collected by every site research coordinator and reviewed the by local principal investigator/physician co-author, what includes but it is not limited to the adverse events (mortality and complications). The statistical analysis, reviewed by the first author, was restricted to continuous data summarized with mean, standard deviation (SD), median, minimum, maximum, first and third quartiles, and number of evaluable observations. The categorical variables were summarized with frequency counts and percentages. Confidence intervals are presented, where appropriate, using the t-distribution for continuous data and Clopper-Pearson Exact method was used for categorical variables. Statistical significance was set at a *P* < 0.05. The sample size was calculated based on the number of cases that all sites could perform during the period of enrollment, on the confidence level of 95% and on the margin error of 5%.

## Results

Procedural and technical successes (primary effectiveness endpoints) were 100% and 97%, respectively (Table 3). Three patients (3%) had incomplete embolization. The secondary effectiveness endpoints (means, ± SD): time to hemostasis was 2:01 ± 0:48 hours, time to ambulation was 3:42 ± 1:36 hours, time to discharge was 12:59 ± 26:35 hours, procedure length was 1:33 ± 0:44 hours, contrast volume procedure was 123.5 ± 63.9 mL, total blood loss > 5 mL was 0% (Table 3). Up to 96.5% of TRAs were obtained using a 5-Fr introducer sheath. Adequate TRA hemostasis was obtained in 100% of cases with the TR Band™.

**Table 3:** Procedural effectiveness endpoints

Primary Effectiveness Endpoints	UFE (n = 70)	PAE (n = 16)	LT (n = 7)	OHT (n = 6)	All Subjects (n = 99)
**Procedure Success**^a^	100%	100%	100%	100%	100%
Radial-to-femoral Conversion	0%	0%	0%	0%	0%
**Technical Success**^b^					
Complete	98.6%	87.5%	100%	100%	97.0%
Incomplete	1.4%	12.5%	0.0%	0.0%	3.0%
**Secondary Effectiveness Endpoints**					
Time to hemostasis^c^(n)	1:52 ± 0.47 (68)	2:14 ± 0.31 (16)	2:52 ± 0.56 (7)	2:13 ± 0.36 (4)	2:01 ± 0:48 (95)
Time to ambulation^c^(n)	3:34 ± 1:36 (69)	3:51 ± 1:30 (16)	4:05 ± 1:36 (7)	4:24 ± 2:07 (5)	3:42 ± 1:36 (97)
Time to discharge^c^(n)	12:48 ± 26:28 (70)	4:43 ± 1:02 (16)	12:41 ± 17:00 (7)	37:34 ± 53:45 (6)	12:59 ± 26:35 (99)
Procedure Length^c^(n)	1:28 ± 0.40 (70)	2:12 ± 0:51 (16)	1:14 ± 0:24 (7)	1:16 ± 0:38 (6)	1:33 ± 0:44 (99)
Contrast volume used mL^c^(n)	119.3 ± 59.8 (70)	149.3 ± 72.0 (16)	93.1 ± 26.8 (7)	100.0 ± 66.6 (6)	123.5 ± 61.9 (99)
Total Blood Loss (> 5 cc)	0%	0%	0%	0%	0% (99)

^a^Procedural success is defined as: completion of the planned procedure without femoral access bailout

^b^Technical success is defined as: delivery of HydroPearl™ to the target vessel until blood stasis was obtained (i.e., complete embolization)

^c^Mean ± SD/ [HH:MM]

The primary safety endpoints up to 30 days showed two (2%) deaths, one secondary to respiratory failure (related to the procedure) and one secondary to progression of liver disease (unrelated to the procedure). Both deaths were unrelated to the TRA or devices used. There was no myocardial infarct, or clinically symptomatic stroke (Table 4). The trans-procedure secondary safety endpoints results were as follows: RA spasm (9.1%), RA dissection (0%), and forearm artery perforation (0%). The use of vasodilators provided prevention of and resolution of vasospasms and there were no cases aborted because of vasospasm. The 30 days TRA-specific adverse events secondary safety endpoints outcomes were: puncture site hematoma (8.1%), symptomatic RA occlusion (0%), hand ischemia (0%), arteriovenous fistula (0%), pseudoaneurysm (0%), distal embolism (0%), finger amputation or limb loss (0%), or necessity of surgical or endovascular intervention post-procedure (0%). Global analysis of AEs within the 30 days follow-up revealed that there were 110 reportable events that occurred in 42/99 (42.4%) patients. There were 12/110 (11%) SAEs, which occurred in 8/99 (8.1%) patients; 7/12 were related to the procedure and 5/12 were unrelated. In addition to the two deaths mentioned above (one related to the procedure), four patients had abdominal pain, and one patient had nausea/vomiting related to the embolization therapy. A total of 103/115 (89.6%) AEs occurred in 41/99 (41.4%) of patients. There were 23/115 AEs that were unrelated to the procedures, which occurred in 18/99 (18.2%) patients. In relation to devices, there were zero SAEs and four AEs (Table 5) (19). Table 6 shows the distribution of procedure settings among the six medical centers that participated in the RAVI registry; 31/99 (31.3%) procedures were done in an office-based lab (OBL), and 68 (68.6%) in a hospital setting.

**Table 4:** Procedural safety endpoints

Primary Safety Endpoints	UFE (***n*** = 70)	PAE (***n*** = 16)	LT (***n*** = 7)	OHT (***n*** = 6)	All Subjects (***n*** = 99)
**SAE within 30 days**					
Death	1.4%^a^	0.0%	14.3%^b^	0.0%	2.0%
Myocardial infarct	0.0%	0.0%	0.0%	0.0%	0.0%
Symptomatic stroke	0.0%	0.0%	0.0%	0.0%	0.0%
Hand Ischemia	0.0%	0.0%	0.0%	0.0%	0.0%
**Secondary Safety Endpoints**					
**Immediate, trans-procedure**					
Radial artery spasm	7.1%	6.3%	28.6%	16.7%	9.1%
Radial artery dissection	0.0%	0.0%	0.0%	0.0%	0.0%
Forearm artery perforation	0.0%	0.0%	0.0%	0.0%	0.0%
**RA-specific complications within 30 days**					
Hematoma at the puncture site^c^	5.7%	12.5%	14.3%	16.7%	8.1%
Symptomatic RA occlusion	0.0%	0.0%	0.0%	0.0%	0.0%
AV Fistula	0.0%	0.0%	0.0%	0.0%	0.0%
Pseudoaneurysm	0.0%	0.0%	0.0%	0.0%	0.0%
Distal embolism	0.0%	0.0%	0.0%	0.0%	0.0%
Finger amputation/loss of limb	0.0%	0.0%	0.0%	0.0%	0.0%
Surgical/endovascular intervention	0.0%	0.0%	0.0%	0.0%	0.0%

^a^Acute hypoxic respiratory failure – not related to technique or device

^b^End stage liver disease – not related to procedure or device

^c^Not clinically relevant

*RA* radial artery

*AV* arteriovenous fistula

**Table 5:** Adverse events within 30-days

Event Type	Number of Events	% of Subjects with events (***n*** = 99)^a^
**Total number of events**	**115**	**42.4%**
Serious/ SAE	**12**	**8.1%**
Non-Serious/AE	**103**	**41.4%**
**Related to Device**	**4**	**4.0%**
Serious/ SAE	**0**^a^	**0.0%**
Non-Serious/ AE	**4**	**4.0%**
**Related to Procedure**	**87**	**36.4%**
Serious/ SAE	**7**^b^	**5.1%**
Non-Serious/ AE	**80**	**34.3%**

Of the 12 serious AEs: ^a^ No serious AE were related to the study device or access site, ^b^7 serious AE events occurring in 5 subjects were related to the study procedure, including: abdominal pain (4), vomiting / nausea (2), hypoxia (1)

**Table 6:** Distribution of cases based on procedure setting

Procedure setting	# cases
Hospital	68/99
Office-based lab	31/99

## Discussion

The RAVI Registry is a prospective, multicenter, open-label study in a cohort of 99 patients who underwent embolization procedures via TRA. The goal was to analyze “real-world” RAVI outcomes and to address some skepticism about the effectiveness and safety of TRA in embolization procedures. Different from TFA, TRA has the potential risk of stroke and hand ischemia. The RAVI registry outcomes show that TRA is safe when a standard technique is used [15]. There was no clinically symptomatic stroke or hand ischemia in 97/99 patients who had follow-up clinic visits in 30 days, with procedures performed by different operators from 6 medical centers. There were 2/99 deaths (2%) within the 30 days of follow-up, only one related to the embolization procedure. This patient had bilateral hydronephrosis and acute renal failure secondary to large fibroids and died of acute hypoxia and multiorgan failure secondary to systemic Methicillin-resistant *Staphylococcus aureus* infection, pulmonary septic emboli, and mitral valve regurgitation. The second death was unrelated to the procedure but to the progression of end-stage liver disease. There were no deaths related to the TRA or to the devices. Other SAEs were abdominal pain in four patients and nausea/vomiting in two patients. These patients required short hospital readmission, and the symptoms were controlled with intravenous medications. All these symptoms were related to post-embolization syndrome.

TRA was also a very effective access for embolization therapies. The procedure success rate was 100%, with 0% of conversion from TRA to TFA. The technical success was also very high (97%). The reasons for the three partial embolizations were as follows: two were partially successful PAEs, one secondary to the inability to access the right prostatic artery, and the second was related to a middle rectal artery branching from the right prostatic artery. In the third case, partial embolization of extremely large fibroids required a repeat UFE. The high effectiveness and safety profile of TRA in the RAVI registry can, at least in part, be explained by the fact that there has been an increased standardization of TRA practice in the last few years. In 2021, Gayed et al. published the Society of Interventional Radiology Quality Improvement Standards on Radial Artery Access [15]. This consensus document from different RAVI experts emphasized important strategies to minimize TRA adverse events, such as: caution on using TRA in elderly patients (> 70 y) with severe aortic arch atherosclerosis; screening patients with Barbeau’s and US exams to evaluate whether the arterial palmar arch is complete, and to check patency and compatibility between the radial artery inner-to-inner wall diameter and the outer diameter of the introducer sheath, respectively. In addition, obtain TRA under US guidance to increase access precision; perform forearm angiography as a roadmap to detect arterial spasm, kinks, and loops and to prevent RA branch perforation; obtain access from the wrist to the descending aorta under fluoroscopic guidance to prevent unnecessary supra-aortic vessels manipulation; use of anticoagulation and vasodilator(s) to minimize the risk of RA thrombosis and spasm, respectively; use “patent hemostasis” concept to prevent bleeding and to reduce the risk of RA occlusion [15]. The relatively recent availability of new and longer devices to support superselective embolizations in the pelvis has also contributed to an exponential growth of TRA in embolization therapy, making TRA the preferential access in an increasing number of medical centers. Another recent national trend captured in the RAVI registry was the shift of procedures typically done in a hospital to an OBL setting. Almost 1/3 of enrolled patients had RAVI performed in an OBL outside the hospital setting. Regarding demographics, most patients in this cohort were female, which is related to the fact that the most common RAVI registry procedure was the embolization of UFEs. Most patients were African American or Caucasian, which is not aligned with the USA racial distribution, but it is with the incidence of pathologies in each racial group, especially uterine fibroids [20]. Hypertension and drug allergies were the most common comorbidities identified, as both are prevalent in the community.

Among the secondary safety endpoints, there was RA spasm in 9.1% of all patients, and it is unclear why it was more prevalent in the group of patients who underwent LT treatment, even though LT had a small sample size. The temporary RA vasospasm was managed and, in most cases, prevented with the use of vasodilator(s) per standard of care and there was no need to convert from TRA to TFA. With adequate devices and techniques [15], there were no RA dissection or forearm arterial perforations. The most common TRA-specific adverse event was self-limited hematomas at the puncture site, which can, at least in part, be explained by using heparin. These patients reported mild discomfort for 3–5 days post-procedure at the puncture site that resolved spontaneously, and no patients reported persistent discomfort, wrist dysfunction or nerve damage at the 30-day follow-up. There was no symptomatic radial artery occlusion, AV fistula, pseudoaneurysm, distal embolization to the digits, finger amputation or need for surgical/endovascular intervention at 30-day follow-up. We speculate that these positive outcomes are partially attributed to the use of US guidance during TRA and to the patent hemostasis concept (associated with an external compression device) to obtain hemostasis at the end of the procedure [16]. There were no surprises in the results of the secondary effectiveness endpoints. The times to hemostasis, ambulation, and discharge showed the expected results. However, the 2-hour mean time to hemostasis could potentially be reduced with standardization of the hemostasis part of the RAVI procedure. This interval showed that it is possible to obtain hemostasis in about 60 minutes even after the administration of a low dose (3–5000 IU, intravenously) of heparin. Also, there are ongoing studies analyzing the safety and efficacy of a 1-hour hemostasis protocol. The global procedure times showed an expected mean of about 90 minutes, with PAE being the RAVI procedure that took the longest. It is speculated that this finding is related to more challenging vascular anatomy and navigation into the prostatic arteries. As demonstrated in previous comparative TFA versus TRA studies in visceral interventions [7–9, 12], safe and effective embolization procedures via TRA required no extra procedure time.

This registry had a few limitations. It is acknowledged that having a brain MRI post-RAVI would have increased the sensitivity to detected silent strokes. It was not included in this registry protocol because it is not part of best clinical practices in either IR or Interventional Cardiology. As 97/99 (98%) patients were asymptomatic during history and physical exam at 30 days follow-up clinic visit, an US examination of the RA puncture site was not ordered to rule out asymptomatic RA occlusion, pseudoaneurysm, or arterio-venous fistula formation. It is anticipated that some cases of radial occlusion could have been detected by an US exam. Ultimately, what matters is that having a positive pre-procedure Barbeau test is associated with complete patency of the arterial palmar arch. Consequently, the ischemia of the thumb and index fingers can be prevented in case of RA occlusion. The authors also recognize an uneven distribution of cases among the participating institutions. As a result of the COVID-19 pandemic, enrollment was slower in some centers compared to others.

## Conclusion

This prospective, multicenter, open-label registry confirmed the high safety profile and effectiveness of radial access in abdominal and pelvic embolization procedures. There were no clinically evident strokes, hand ischemia or access-related serious adverse events at 30 days follow-up. Radial access safety and effectiveness suggest that TRA can be used as the primary access in embolization procedures.
